# Supplementary material for: Identification of Freezing-Responsive microRNAs and Their Targets in Chinese Jujube by Small RNA and Degradome Sequencing
Source: Int J Mol Sci. 2026 Sep 4;27(17):7912. doi: 10.3390/ijms27177912 (PMC13566890; doi:10.3390/ijms27177912)
Supplement: Supplementary file 1 [file ijms-27-07912-s001.zip › Supplementary Material S2/novel_miR_231.pdf]

Provisional ID	:	novel_miR_231
Score total	:	0.6
Score for star read(s)	:	-1.3
Score for read counts	:	0
Score for mfe	:	0.3
Score for randfold	:	1.6
Score for cons. seed	:	
Total read count	:	104
Mature read count	:	65
Loop read count	:	39
Star read count	:	0

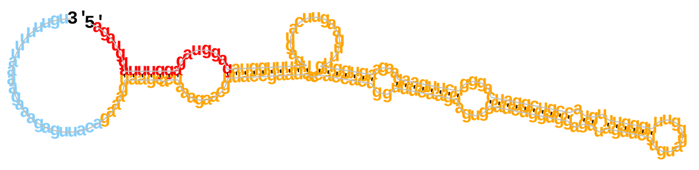

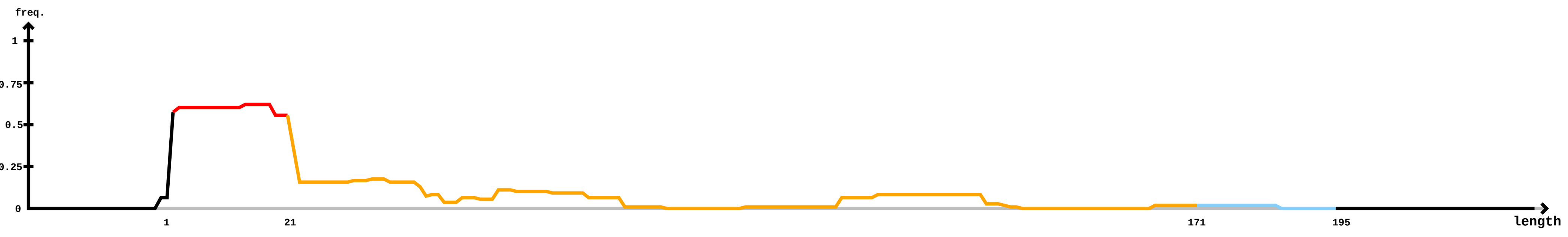

Mature Star

```
5'- gcugauuuugguaagaagaugcagauggauuuggagauggagaugguuuaugaguacuugaaguuuguggugaagaagaaguucugggacuaggcugccauguuuggguuugggaugugcuugauugaggugguucuaggugaagaacuuuggucaccaccaauaagccauugcaagaaaucacgaaugaaagacauugagaaagaaaauuuuuuuguauuuuguuuaaagacugaauaaauagcaucacaa -3'
    ((((..((((((((.((((((((((.((((((...((((....(.((((.(((...((((..(((((((((((....((((((((....((((((((((.((.((((((.........)))))).)).))))))))))....))))))))..)))))))....))))).....))))...))).)))).)....))))........))))))))))))))).)).....))))))....))))......
```

exp	reads	mm	sample
gcagauggauuuggagaug	6	0	LCK-3
gcagauggauuuggCgaug	1	1	LCK-3
agauggauuuggagaugAag	1	1	LCK-2
agauggauuuggagauggag	22	0	LCK-2
agauggauuuggagauggag	32	0	LCK-1
gauggauuuggagauggagU	3	1	LT-2
agauggagaugguuuaugaguacu	2	0	JCK-2
ugguuuaugaguacuugaag	4	0	LT-2
ugguuuaugaguacuugaagu	6	0	LCK-3
ugguuuaugaguacuugaaguuug	5	0	LT-2
aguacuugaaguuugugguga	1	0	LT-1
acuugaaguuuguggugaagaaga	1	0	LT-1
uuuguggugaagaagaaguucu	1	0	LT-1
uguggugaagaagaaguuc	1	0	LT-3
ugaagaagaaguucugggacu	3	0	LT-3
agaaguucugggacuaggcug	6	0	LCK-3
ugggacuaggcugccauguu	1	0	LT-3
ugcuugauugaggugguucuagg	1	0	LT-1
ucuaggugaagaacuuuggucacc	4	0	LCK-3
ucuaggugaagaacuuuggucacc	2	0	LT-3
ugaagaacuuuggucaccacc	1	0	LT-3
ugaagaacuuuggucaccacca	1	0	LT-3
ugaagaacuuuggucaccaccaau	1	0	LT-1
augaaagacauugagaaagaa	2	0	LT-1
